# Clinical characteristics and risk factors for 28-day mortality in critically ill patients with COVID-19: a retrospective cohort study

**DOI:** 10.3906/sag-2104-356

**Published:** 2021-10-21

**Authors:** Mehmet Nuri YAKAR, Begüm ERGAN, Bişar ERGÜN, Murat KÜÇÜK, Ali CANTÜRK, Mahmut Cem ERGON, Naciye Sinem GEZER, Erdem YAKA, Bilgin CÖMERT, Ali Necati GÖKMEN

**Affiliations:** 1 Department of Anaesthesiology and Reanimation, Division of Intensive Care, Faculty of Medicine, Dokuz Eylül University, İzmir Turkey; 2 Department of Chest Diseases, Division of Intensive Care, Faculty of Medicine, Dokuz Eylül University, İzmir Turkey; 3 Department of Internal Medicine, Division of Intensive Care, Faculty of Medicine, Dokuz Eylül University, İzmir Turkey; 4 Department of Radiology, Faculty of Medicine, Dokuz Eylül University, İzmir Turkey; 5 Department of Medical Microbiology, Faculty of Medicine, Dokuz Eylül University, İzmir Turkey; 6 Department of Neurology, Division of Intensive Care, Faculty of Medicine, Dokuz Eylül University, İzmir Turkey; 7 Department of Internal Medicine and Critical Care, Medicana International Hospital, İzmir Turkey

**Keywords:** COVID-19, critical care, tomography, mortality, risk factors, SARS-CoV-2

## Abstract

**Background:**

To date, the coronavirus disease 2019 (COVID-19) caused more than 2.6 million deaths all around the world. Risk factors for mortality remain unclear. The primary aim was to determine the independent risk factors for 28-day mortality.

**Materials and Methods:**

In this retrospective cohort study, critically ill patients (≥ 18 years) who were admitted to the intensive care unit due to COVID-19 were included. Patient characteristics, laboratory data, radiologic findings, treatments, and complications were analyzed in the study.

**Results:**

A total of 249 patients (median age 71, 69.1% male) were included in the study. 28-day mortality was 67.9% (n = 169). The median age of deceased patients was 75 (66–81). Of them, 68.6% were male. Cerebrovascular disease, dementia, chronic kidney disease, and malignancy were significantly higher in the deceased group. In the multivariate analysis, sepsis/septic shock (OR, 15.16, 95% CI, 3.96–58.11, p < 0.001), acute kidney injury (OR, 4.73, 95% CI, 1.55–14.46,p = 0.006), acute cardiac injury (OR, 9.76, 95% CI, 1.84–51.83, p = 0.007), and chest CT score higher than 15 (OR, 4.49, 95% CI, 1.51-13.38, p = 0.007) were independent risk factors for 28-day mortality.

**Conclusion:**

Early detection of the risk factors and the use of chest CT score might improve the outcomes in patients with COVID-19.

## 1. Introduction

The first definition of coronavirus disease 2019 (COVID-19) as unexplained pneumonia was reported on December 8, 2019, in Hubei Province, Wuhan, China [1]. The disease spread rapidly, and, as of April 11th, 2021, almost 135 million patients had tested positive for the disease and around 3 million deaths have been associated with the disease [2]. Several studies have defined patient characteristics and outcomes, but risk factors for mortality remain unclear. Although the outcomes of the studies vary, older age, male sex, obesity, hypertension, type 2 diabetes mellitus, active malignancy, coronary artery disease, a low PaO_2 _/ FiO_2_ ratio on admission, the need for invasive mechanical ventilation (IMV), high D-dimer and C-reactive protein (CRP) levels were defined as risk factors for mortality in different patient cohorts [3–8].

The rapid spread of COVID-19 increased the stress on limited intensive care sources in Turkey as well as around the world. However, it became more difficult to prevent disease progression and reduce mortality due to the uncertainty of effective therapies. There are limited data for critically ill patients with COVID-19 from Turkey, and we designed the present study to evaluate possible undefined risk factors for mortality in patients with severe COVID-19 infection. 

In the present study, we aimed to assess patients with laboratory or clinically confirmed COVID-19 admitted to the intensive care unit (ICU) and to define the independent risk factors related to 28-day mortality.

## 2. Methods

### 2.1. Study population

In this retrospective, single-center study, we evaluated the patients treated in our center’s ICUs due to COVID-19 between March and November 2020. The local ethics committee (2020/09-39/09.11.2020), and the Turkish Ministry of Health approved the study. Patients with length of ICU stay shorter than 24 h were excluded. Written informed consent was waived due to the study design. 

### 2.2. Data collection 

Patient characteristics, epidemiologic, laboratory, and radiologic data were collected from electronic records and each patient’s paper charts. Demographic data, comorbidities, laboratory data including blood tests, and microbiological cultures, patient characteristics (body mass index, smoking history, exposure history, use of immunosuppressive drug), symptoms, and signs for COVID-19, Acute Physiology and Chronic Health Evaluation II (APACHE II) score, Charlson comorbidity index (CCI), Sequential Organ Failure Assessment (SOFA) score calculated on the ICU admission day were recorded. 

The day that the first symptom was noticed considered the onset of the disease. Hospitalization characteristics including emergency or in-patient admission, the onset of symptoms to hospital/ICU admission, length of hospital/ICU stay, time from hospital admission to ICU admission; all therapies, including antiviral therapies, corticosteroid therapy, convalescent plasma transfusion, antithrombotic therapies, immunomodulator therapies, renal replacement therapy (RRT), respiratory support modalities were recorded. Patient’s medical records were screened for possible complications as sepsis/septic shock, acute cardiac injury, acute kidney injury (AKI), ventilator-associated pneumonia (VAP), pulmonary thromboembolism, new-onset arrhythmia, and cardiopulmonary resuscitation. AKI was identified according to KDIGO criteria [9]. Cardiac injury was defined if the high-sensitive troponin I levels were above the 99th percentile higher reference limit [10]. Sepsis and septic shock were defined according to current guidelines [11]. The diagnosis of VAP depends on a previous study [12]. Chest computed tomography (CT) scans were classified as (I) negative, (II) typical appearance, (III) atypical appearance, and (IV) indeterminate appearance for COVID-19 pneumonia according to an expert consensus statement [13]. A scoring system was used to quantitatively estimate the pulmonary involvement of the CT scans, which demonstrates a typical and indeterminate appearance for COVID-19 [14]. All lung lobes were scored visually from 0 to 5 points. The total CT score was ranged between 0 to 25. Normal view with no involvement scored as 0 points. Involvement levels scored as follows: less than 5%, 1 point; 5%–25%, 2 points; 26%–49%, 3 points; 50%–75%, 4 points, and more than 75%, 5 points. Atypical CT scans were not scored since radiologic findings were incompatible with COVID-19. Two experienced radiologists performed the image analysis. 

### 2.3. Statistical analysis

The primary outcome of the study was to define independent risk factors for 28-day mortality in critically ill patients with COVID-19. Secondary outcome was to determine clinical characteristics, laboratory data, complications, and the predictive value of chest CT score for 28-day mortality. The SPSS Statistics software (Statistical Package for the Social Sciences Version 24.0; IBM Corporation, Armonk, NY, USA) was used to perform statistical analysis of the data. Continuous variables were stated as the median and interquartile range (IQR) and analyzed using the Mann–Whitney U test. Categorical variables were stated as counts and percentages and analyzed by chi-square, or Fisher exact test. To evaluate the intra-rater agreement for chest CT scoring, intra-class correlation coefficient (ICC) was used with a 95% confidence interval (CI). The independent effect of each factor on 28-day mortality was determined using logistic regression analysis. A rational method was used to select a subset of clinically significant covariates. An odds ratio (OR) with a 95% CI was noticed for each independent factor. Statistical significance was regarded as a two-tailed *p*-value lower than 0.05.

## 3. Results

### 3.1. Patient characteristics

A total of 249 patients (69.1%, male) were included in the study, and patients were divided into two groups: recovered (n = 80) and deceased (n = 169) (Figure). 

**Figure:**
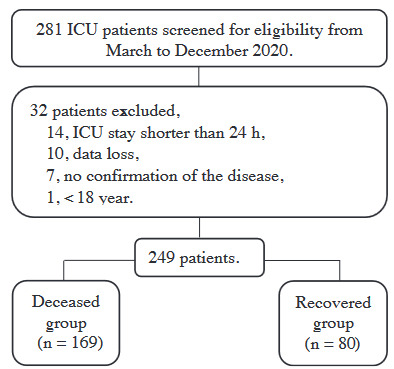
Flowchart of the study.

The mortality rate was 67.9%. The median age was 71 (61–80) years (Table 1). The median APACHE II score, CCI, and SOFA score were 20 (12–27), 5 (3–7), and 5 (3–7), respectively. Most of the patients had at least one or more coexisting comorbidities. Hypertension, diabetes mellitus, and coronary artery disease were the most common comorbidities. On hospital admission, dyspnea, dry cough, and fever were the most common symptoms. In-patient admissions were more often compared with the others.

**Table 1:** Baseline characteristics of the patients.

		28-day status		
Clinical Characteristics	All cases(n=249)	Recovered group (n=80)	Deceased group (n=169)	P Value
Age, years	71 (61-80)	63 (53-71)	75 (66-81)	< 0.001
Sex				
Male	172 (69.1)	24 (30.0)	116 (68.6)	0.88
Female	77 (30.9)	56 (70.0)	53 (31.4)	
BMI, kg/m2	26.1 (23.8-29.3)	27.0 (24.0-29.9)	26.0 (23.3-29.0)	0.21
APACHE II score	20 (12-27)	14 (9-22)	23 (15-28)	< 0.001
SOFA Score a	5 (3-7)	4 (2-5)	6 (4-8)	< 0.001
CCI	5 (3-7)	2 (1-5)	6 (4-8)	< 0.001
Smoking history	55 (22,1)	13 (16.3)	42 (24.9)	0.14
Exposure history	87 (34.9)	26 (32.5)	61 (36.1)	0.67
Immunosuppressive drug use	15 (6.0)	5 (6.3)	10 (5.9)	1.00
RT-PCR test positivity	222 (89.2)	66 (82.5)	156 (92.3)	0.028
Comorbidities	215 (86.3)	61 (76.3)	154 (91.1)	0.003
Hypertension	178 (71.5)	52 (65.0)	126 (74.6)	0.13
Diabetes mellitus (Type 2)	96 (38.6)	29 (36.3)	67 (39.6)	0.68
Coronary artery disease	64 (25.7)	15 (18.8)	49 (29.0)	0.09
Congestive heart failure	35 (14.1)	6 (7.5)	29 (17.2)	0.05
Chronic kidney disease	38 (15.3)	6 (7.5)	32 (18.9)	0.023
Dementia	30 (12.0)	3 (3.8)	27 (16.0)	0.006
COPD	29 (11.6)	9 (11.3)	20 (11.8)	1.00
Malignancy b	25 (10.0)	3 (3.8)	22 (13.0)	0.024
Cerebrovascular diseases	17 (6.8)	0 (0.0)	17 (10.1)	0.002
Hyperlipidemia	16 (6.4)	6 (7.5)	10 (5.9)	0.59
Parkinson’s disease	7 (2.8)	1 (1.3)	6 (3.6)	0.44
Thromboembolic event history	5 (2.0)	2 (2.5)	3 (1.8)	0.66
Chronic liver disease	1 (0.4)	0 (0.0)	1 (0.6)	1.00
Signs and symptoms				
Dyspnea	172 (69.1)	49 (61.3)	123 (72.8)	0.08
Dry cough	122 (49.0)	41 (51.3)	81 (47.9)	0.68
Fever	118 (47.4)	41 (51.3)	77 (45.6)	0.42
Fatigue	76 (30.5)	32 (40.0)	44 (26.0)	0.028
Myalgia	36 (14.5)	18 (22.5)	18 (10.7)	0.020
Expectoration	32 (12.9)	3 (3.8)	29 (17.2)	0.002
Nausea/Vomiting	29 (11.6)	7 (8.8)	22 (13.0)	0.40
Anorexia	24 (9.6)	3 (3.8)	21 (12.4)	0.037
Chest pain	20 (8.0)	8 (10.0)	12 (7.1)	0.46
Sore throat	16 (6.4)	8 (10.0)	8 (4.7)	0.16
Diarrhea	11 (4.4)	3 (3.8)	8 (4.7)	1.00
Loss of smell or taste	10 (4.0)	4 (5.0)	6 (3.6)	0.73
Headache	8 (3.2)	4 (5.0)	4 (2.4)	0.27
Palpitations	7 (2.8)	2 (2.5)	5 (3.0)	1.00
Abdominal pain	5 (2.0)	0 (0.0)	5 (3.0)	0.18
Hospitalization characteristics				
Onset of symptom to,				
Hospital admission, days	3 (1-6)	3 (2-6)	3 (1-5)	0.08
ICU admission, days	6 (3-10)	8 (4-10)	6 (3-10)	0.21
Admission,				
In-patient	166 (66.7)	57 (71.3)	109 (64.5)	0.32
Emergency	83 (33.3)	23 (28.8)	60 (35.5)	
Length of Hospital stay before ICU admission, days	2 (1-5)	2 (1-5)	2 (1-5)	0.58
Length of ICU stay, days	8 (4-14)	6 (4-13)	9 (4-14)	0.25
Length of hospital stay, days	14 (9-21)	17 (12-25)	13 (8-19)	< 0.001

All values are expressed as numbers (percentages) or median (interquartile range).Abbreviations: BMI, body mass index; APACHE II, Acute Physiology and Chronic Health Evaluation II; SOFA score, The Sequential Organ Failure Assessment Score; CCI, Charlson Comorbidity Index; RT-PCR test; real time – polymerase chain reaction test; COPD, chronic obstructive pulmonary disease; ICU, intensive care unit.a Calculated on the day of ICU admission.

Compared with the recovered group, deceased patients were significantly older and sicker (with higher APACHE II, SOFA and CCI scores) and had higher rates of comorbidities (including cerebrovascular disease, dementia, chronic kidney disease and malignancy). Symptoms as expectoration and anorexia were observed more in this group as well. The median length of ICU stay was three days longer in the deceased group than the recovered group, but not statistically significant.

### 3.2. Laboratory data and chest CT findings

In this study, higher median values of white blood cell (12.4 [8.7–16.1] vs. 9.3 [7.6–12.3] x 10^3^/µL, *p *= 0.001), neutrophil counts (10.5 [7.2–14.6] vs. 8.3 [6.2–11.0] × 10^3^/µL, *p *= 0.001), as well as higher median levels of blood urea nitrogen (38.0 [27.0–55.4] vs. 24.0 [18.0–31.5] mg/dL, *p *< 0.001), and creatine (1.20 [0.82–1.87] vs. 0.93 [0.73–1.10] mg/dL, *p *< 0.001) were notable in the deceased group than the recovered group (Table 2). The median value of laboratory findings such as C-reactive protein (164 [80-250] vs. 124 [85-197] mg/L, *p *= 0.030), ferritin (640 [377–1134] vs. 482 [255–1061] ng/mL, *p *= 0.013), and procalcitonin (0.48 [0.19–1.77] vs. 0.15 [0.08–0.32] ng/mL, *p *< 0.001), high-sensitive troponin I (38.0 [15.0–313.0] vs. 11.5 [6.7–40.0] ng/L, *p *< 0.001), plasma brain natriuretic peptide (120 [46–379] vs. 51 [27–172] pg/mL, *p *< 0.001), and D-dimer (2.0 [1.2–3.9] vs. 1.0 [0.5–2.0] µg/mL, *p *< 0.001) were higher in the deceased group than the recovered group. 

**Table 2:** Laboratory data, microbiological culture results, and chest CT findings.

		28-day status		
Laboratory Data a	All cases (n=249)	Recovered group (n=80)	Deceased group (n=169)	P Value
WBC, x 103/µL	11.2 (8.1-15.0)	9.3 (7.6-12.3)	12.4 (8.7-16.1)	0.001
Neutrophil, x 103/µL	9.6 (6.8-13.8)	8.3 (6.2-11.0)	10.5 (7.2-14.6)	0.001
Hemoglobin, g/dL	12.5 (11.0-13.8)	12.9 (11.6 -13.9)	12.3 (10.4-13.8)	0.05
Lymphocyte, x 103/µL	0.5 (0.3-0.9)	0.5 (0.3-0.8)	0.5 (0.3-1.0)	0.61
Lymphocyte, %	5.6 (3.1-8.9)	6.1 (4.1-10.3)	5.2 (2.8-8.4)	0.10
Platelet, x 103/µL	252.0 (172.0-334.5)	273.5 (184.3-341.8)	243.0 (167.5-332.0)	0.26
BUN, mg/dL	31.0 (23.0-50.0)	24.0 (18.0-31.5)	38.0 (27.0-55.4)	< 0.001
Creatinine, mg/dL	1.00 (0.80-1.60)	0.93 (0.73-1.10)	1.20 (0.82-1.87)	< 0.001
Total Bilirubin, mg/dL	0.8 (0.6-1.14)	0.7 (0.6-1.1)	0.9 (0.7-1.2)	0.22
CRP, mg/L	152 (83-228)	124 (85-197)	164 (80-250)	0.030
AST, U/L	52 (37-89)	51 (34-77)	54 (39-93)	0.13
ALT, U/L	37 (24-63)	40 (26-63)	36 (23-62)	0.14
LDH, U/L	550 (421-706)	495 (389-646)	570 (452-761)	0.006
Ferritin, ng/mL	612 (345-1118)	482 (255-1061)	640 (377-1134)	0.013
HS Troponin I, ng/L	27.0 (9.6-142.5)	11.5 (6.7-40.0)	38.0 (15-313)	< 0.001
D-Dimer, µg/mL	1.5 (0.9-3.6)	1.0 (0.5-2.0)	2.0 (1.2-3.9)	< 0.001
Procalcitonin, ng/mL	0.34 (0.13-1.12)	0.15 (0.08-0.32)	0.48 (0.19-1.77)	< 0.001
BNP (plasma), pg/mL	87 (39-307)	51 (27-172)	120 (46-379)	< 0.001
Arterial blood gas analysis a				
pH	7.41 (7.32-7.47)	7.44 (7.39-7.48)	7.37 (7.29-7.46)	< 0.001
PaCO2, mmHg	35 (30-42)	33 (30-38)	36 (30-44)	0.08
PaO2, mmHg	65 (54-79)	68 (58-80)	63 (53-78)	0.09
HCO3, mmol/L	22.8 (20-25)	24.0 (21.4-26.0)	22.0 (18.2-25.0)	< 0.001
Lactate, mmol/L	2.0 (1.4-3.0)	1.8 (1.3-2.4)	2.1 (1.4-3.3)	0.001
SO2, %	92 (87-95)	93 (90-96)	91 (86-94)	0.002
FiO2, %	60 (50-60)	50 (50-60)	60 (50-60)	< 0.001
PaO2 / FiO2	116 (97-152)	129 (108-162)	112 (91-144)	0.001
PaO2 / FiO2 ≤ 100	75 (30.1)	12 (15.0)	63 (37.3)	< 0.001
PaO2 / FiO2 ≤ 200	217 (87.1)	64 (80.0)	153 (90.5)	0.026
100< PaO2 / FiO2 ≤ 200	142 (57.0)	52 (65.0)	90 (53.3)	0.10
200< PaO2 / FiO2 ≤ 300	21 (8.4)	11 (13.8)	10 (5.9)	0.05
PaO2 / FiO2 > 300	11 (4.4)	5 (6.3)	6 (3.6)	0.34
Blood culture b	64 (25.7)	11 (13.8)	53 (31.4)	0.003
Acinetobacter spp.	34 (13.7)	8 (10.0)	26 (15.4)	0.32
Klebsiella spp.	13 (5.2)	2 (2.5)	11 (6.5)	0.23
Enterobacter spp.	13 (5.2)	3 (3.8)	10 (5.9)	0.56
Pseudomonas spp.	5 (2.0)	1 (1.3)	4 (2.4)	1.00
Respiratory sample culture	96 (38.6)	13 (16.3)	83 (49.1)	< 0.001
Acinetobacter spp.	47 (18.9)	8 (10.0)	39 (23.1)	0.015
Klebsiella spp.	20 (8.0)	1 (1.3)	19 (11.2)	0.005
Pseudomonas spp.	7 (2.8)	2 (2.5)	5 (3.0)	1.00
Enterobacter spp.	2 (0.8)	0 (0.0)	2 (1.2)	1.00
Urine Culture	70 (28.1)	19 (23.8)	51 (30.2)	0.37
Acinetobacter spp.	5 (2.0)	2 (2.5)	3 (1.8)	0.66
Klebsiella spp.	10 (4.0)	3 (3.8)	1 (1.3)	1.00
Pseudomonas spp.	3 (1.2)	1 (1.3)	2 (1.2)	1.00
Enterobacter spp.	11 (4.4)	3 (3.8)	8 (4.7)	1.00
Escherichia coli	20 (8.0)	6 (7.5)	14 (8.3)	1.00
Candida spp.	23 (8.2)	7 (8.8)	16 (9.5)	1.00
Chest CT Score c	15 (10-20)	13 (9-19)	16 (12-20)	0.05
Chest CT Score > 15	106 (42.6)	27 (33.8)	79 (46.7)	0.034
Chest CT Compatibility				
Typical	194 (77.9)	63 (78.8)	131 (77.5)	0.17
Atypical	27 (10.8)	4 (5.0)	23 (13.6)	0.08
Indeterminate appearance	5 (2.0)	1 (1.3)	4 (2.4)	1.00
Negative	4 (1.6)	2 (2.5)	2 (1.2)	0.59
No CT scan	19 (7.6)	10 (12.5)	9 (5.3)	0.71

All values are expressed as numbers (percentages) or median (interquartile range).Abbreviations: CT, computed tomography; WBC, white blood cell; BUN, blood urea nitrogen; CRP, C-reactive protein; AST, aspartate transaminase; ALT, alanine transaminase; LDH, lactate dehydrogenase; HS Troponin I, high-sensitive troponin I; BNP, brain natriuretic peptide; PaCO2, partial pressure of arterial carbon dioxide; PaO2, partial pressure of arterial oxygen; SO2, arterial oxygen saturation; FiO2a Tested on the day of ICU admission.b Collected percutaneously or via a central vein. c Performed on the day of hospital admission.

In the deceased group, the median values of pH (7.37 [7.29–7.46] vs. 7.44 [7.39–7.48], *p *< 0.001), PaO_2 _/ FiO_2_ ratio (112 [91–144] vs. 129 [108–162], *p *= 0.001), HCO_3_ (22.0 [18.2–25.0] vs. 24.0 [21.4–26.0] mmol/L, *p *< 0.001), oxygen saturation on ICU admission (91 [86–94] vs. 93 [90–96], *p *= 0.002) were lower than the recovered group. Patients had a lower PaO_2 _/ FiO_2_ ratio than 200 (153 [90.5%] vs. 64 [80.0%], *p *= 0.026), and 100 (63 [37.3%] vs. 12 [15.0%] *p *< 0.001), and higher median values of lactate (2.1 [1.4–3.3] vs. 1.8 [1.3–2.4] mmol/L, respectively, *p *= 0.001) were more frequent in the deceased group than the recovered group.

Analysis of the bloodstream infection, VAP, and urinary tract infection showed that the number of patients with one or higher positive blood cultures (53 [31.4%] vs. 11 [13.8%], respectively, *p *= 0.003), and respiratory sample cultures (83 [49.1%] vs. 13 [16.3%], respectively, *p *< 0.001); and tracheal aspirate cultures including *Acinetobacter* spp. (39 [23.1%] vs. 8 [10.0%], *p *= 0.015) and *Klebsiella* spp. (19 [11.2%] vs. 1 [1.3%], *p *= 0.005) were significantly high in the deceased group than the recovered group. 

Of all, 230 patients had a chest CT scan performed on hospital admission. Chest CT scans were reevaluated by two radiologists to calculate the CT scores. ICC revealed a high interrater reliability between raters (ICC: 0.995; 95% CI, 0.991–0.998). Chest CT scan compatibility for COVID-19 pneumonia distributed as typical (77.9%), atypical (10.8%), indeterminate appearance (2.0%), and negative (1.6%). The median chest CT score was 15 (10–20). Although the median chest CT score was higher in the deceased group than the recovered group (16 [12–20] vs. 13 [9–19], respectively), there was no statistical significance between the groups (*p *= 0.05). Additionally, participants divided into two groups referring to the median chest CT score and patients who had a chest CT score more than 15 were significantly higher in the deceased group than the recovered group (79 [46.7%] vs. 27 [33.8%], *p *= 0.034).

### 3.3. Treatments and complications

Most patients received antiviral therapies (favipiravir [96.0%], hydroxychloroquine [21.7%]), corticosteroid therapy (corticosteroid [78.3%], pulse corticosteroid [40.6%]) depending on current national treatment guidelines at the time of hospitalization (Table 3). Tocilizumab was given to 25 (10.0%) patients. Of all cases, 70 (28.1%) patients were transfused different amounts of convalescent plasma. Favipiravir treatment was significantly high in the recovered group than the deceased group (100% vs. 94.1%, respectively, *p *= 0.033). RRT (34.3% vs. 8.8%, *p *< 0.001) and vasopressor use (84.6% vs. 27.5%, *p *< 0.001) were more common in the deceased group than the recovered group. Respiratory support modalities distributed as conventional oxygen therapy (COT) (8.0%), high flow nasal cannula (HFNC) (36.1%), noninvasive mechanical ventilation (NIMV) (17.3%), invasive mechanical ventilation (IMV) (77.1%), extracorporeal membrane oxygenation (ECMO) (1.2%). HFNC was more frequently used in the recovered group than the deceased group (52.5% vs. 28.4%, *p *< 0.001), and patients in the deceased group more frequently needed IMV than the recovered group (97.6% vs. 33.8%, *p *< 0.001). Patients who did not need any respiratory support except COT were significantly higher in the recovered group than the deceased group (22.5% vs. 1.2%, *p *< 0.001).

**Table 3:** Treatments and Complications of the patients.

		28-day status		
	All cases (n=249)	Recovered group (n=80)	Deceased group (n=169)	P Value
Treatment				
Favipiravir	239 (96.0)	80 (100.0)	159 (94.1)	0.033
Hydroxychloroquine	54 (21.7)	16 (20.0)	38 (22.5)	0.74
LMWH	237 (95.2)	77 (96.3)	160 (94.7)	0.76
ASA	193 (77.5)	60 (75.0)	133 (78.7)	0.52
Dipyridamole	152 (61.0)	42 (52.5)	110 (65.1)	0.07
Corticosteroidsa	195 (78.3)	61 (76.3)	134 (79.3)	0.62
Pulse corticosteroidb	101 (40.6)	29 (36.3)	72 (42.6)	0.41
Tocilizumab	25 (10.0)	10 (12.5)	15 (8.9)	0.38
Convalescent plasma	70 (28.1)	28 (35.0)	42 (24.9)	1.00
Use of vasopressorc	165 (66.3)	22 (27.5)	143 (84.6)	< 0.001
RRT	65 (26.1)	7 (8.8)	58 (34.3)	< 0.001
Respiratory Support				
COT	20 (8.0)	18 (22.5)	2 (1.2)	< 0.001
HFNC	90 (36.1)	42 (52.5)	48 (28.4)	< 0.001
NIMV	43 (17.3)	12 (15.0)	31 (18.3)	0.52
IMV	192 (77.1)	27 (33.8)	165 (97.6)	< 0.001
ECMO	3 (1.2)	0 (0.0)	3 (1.8)	0.23
Complications				
Sepsis/septic shock	166 (66.7)	18 (22.5)	148 (87.6)	< 0.001
AKI	139 (55.8)	14 (17.5)	125 (74.0)	< 0.001
VAP	118 (47.4)	16 (20.0)	102 (60.4)	< 0.001
Acute cardiac injury	60 (24.1)	6 (7.5)	54 (32.0)	< 0.001
New-onset arrhythmia	50 (20.1)	10 (12.5)	40 (23.7)	0.043
CPR	9 (3.6)	2 (2.5)	7 (4.1)	0.72
PTE	5 (2.0)	3 (3.8)	2 (1.2)	0.33

All values are expressed as numbers (percentages) or median (interquartile range).Abbreviations: LMWH, low molecular weight heparin; ASA, acetylsalicylic acid; RRT, renal replacement therapy; COT, conventional oxygen therapy; HFNC, high flow nasal cannula; NIMV, noninvasive mechanical ventilation; IMV, invasive mechanical ventilation; ECMO, extracorporeal membrane oxygenation; AKI, acute kidney injury; VAP, ventilator-associated pneumonia; CPR, cardiopulmonary resuscitation; PTE, pulmonary thromboembolism.a Intravenous methylprednisolone 0.5–1 mg/kg/day.b Intravenous methylprednisolone > 250 mg/day.c

The most common complications among 249 patients were sepsis/septic shock (66.7%), AKI (55.8%), VAP (47.4%), acute cardiac injury (24.1%), new-onset arrhythmia (20.1%). Of these complications, sepsis/septic shock (87.6% vs. %22.5, *p *< 0.001), AKI (74.0% vs. 17.5%, *p *< 0.001), VAP (60.4% vs. 20.0%, *p *< 0.001), acute cardiac injury (32.0% vs. 7.5%, *p *< 0.001), and new-onset arrhythmia (23.7% vs. 12.5%, *p *= 0.043) were particularly higher in the deceased group than the recovered group.

### 3.4 Multivariate model for independent risk factors for mortality

Logistic regression analysis was conducted to define the independent risk factors for 28-day mortality (Table 4). The model was built by considering patient characteristics such as sex, APACHE II score and chest CT score > 15, comorbidities including malignancy and dementia, and complications, which are also risk factors for mortality, such as AKI, VAP, sepsis/septic shock, and acute cardiac injury. The analysis determined the independent risk factors for 28-day mortality as follows: sepsis/septic shock (OR, 15.16, 95% CI, 3.96–58.11, *p *< 0.001); AKI (OR, 4.73, 95% CI, 1.55–14.46, *p *= 0.006); CT score higher than 15 on hospital admission (OR, 4.49, 95% CI, 1.51–13.38, *p *= 0.007); and acute cardiac injury (OR, 9.76, 95% CI, 1.84–51.83, *p *= 0.007). 

**Table 4:** Logistic regression analysis for independent risk factors of 28-day mortality in critically ill patients with COVID-19.

	OR (95% CI)	P Value
Sepsis/Septic Shock	15.16 (3.96–58.11)	< 0.001
AKI	4.73 (1.55–14.46)	0.006
Acute cardiac injury	9.76 (1.84–51.83)	0.007
VAP	2.23 (0.66–7.61)	0.20
CT Score > 15	4.49 (1.51–13.38)	0.007
APACHE II score	1.02 (0.95–1.10)	0.53
Dementia	3.94 (0.35–40.28)	0.26
Malignancy	1.20 (0.18–8.05)	0.85
Sex	1.45 (0.49–4.33)	0.51

Abbreviations: OR, odds ratio; AKI, acute kidney injury; VAP, ventilator-associated pneumonia; CT score, computed tomography score; APACHE II, Acute Physiology and Chronic Health Evaluation II.

## 4. Discussion

In the present study, we wanted to assess critically ill patients with clinically or laboratory-confirmed COVID-19. We determined the mortality with a rate of 67.9% and found that a chest CT score > 15 on hospital admission and complications including sepsis/septic shock, AKI, and acute cardiac injury independently increase the risk of death.

The mortality rate is higher than expected however we believe the study population consisted of many frail elderly high-risk patients with a high prevalence of organ dysfunction that occurred due to complications as sepsis/septic shock, AKI, acute cardiac injury. Moreover, a high proportion of the patients had PaO_2 _/ FiO_2_ ratio lower than 200 on ICU admission (87.1%) with high intubation rate (77.1%). A case series from the USA revealed high mortality with a rate of 67% as well as this present study [15]. In the case series, the median age was 70 (43–92) years and 85.7% of the patients had at least one or more comorbidities especially CKD, congestive heart failure, diabetes mellitus, and COPD. Of them, 57.1% had severe ARDS, but the median PaO_2_/FiO_2_ ratio was higher compared with this presented study (169 [69–492] vs. 116 [97–152] mmHg). Additionally, complications including cardiomyopathy (33.3%), acute kidney failure (19.1%), and acute hepatic injury (14.3%) were notable. Beyond all, the small number of patients (n=21) in the case series was the main limitation to generalize the results.

In critically ill patients with COVID-19, 28-day mortality varies among studies ranging between 35.4%–67% [5,7,8,10,15,16]. In a case series from China, 28-day ICU mortality was 39% but reached 97% in the subgroup of patients requiring IMV [17]. Another study reported 88.1% ICU mortality in patients who received IMV [18]. Conversely, in a case series with a high proportion of patients who needed IMV (79%), 28-day mortality was 39% [16]. This wide variability may be in consequence of various factors such as patient characteristics, disease severity in different populations, the spreading rate of the disease, the stress on healthcare systems, ICU admission practices and follow-up time in the studies.

In literature, there is limited data for the relationship between involvement in lungs and mortality in COVID-19. In this study, the higher points of the chest CT score were associated with poor prognosis. We have found that a chest CT score > 15 on hospital admission independently increases the risk of death. Likewise, a recent study revealed that a CT score ≥ 18 is highly predictive of short-term mortality in patients with COVID-19 [19]. Additionally, the chest CT score correlates with age, oxygen requirement, and disease progression [19–21]. As a result, we suggest that the chest CT score calculated on the day of hospital admission has a remarkable value owing to present a prediction for the disease progression and 28-day mortality.

Organ impairments such as cardiac or renal injury, and coinfections causing sepsis/septic shock were found as another independent risk factors for 28-day mortality in this study. The relationship between organ failure and increased mortality is usual in ICU. However, acute kidney injury, cardiac injury, and secondary infections are the conditions that should be prevented or needed close monitoring in critically ill patients with COVID-19. In the laboratory data, findings indicating infection, inflammation, myocardial injury, or abnormal cardiac function, as well as abnormal renal function and fluid overload were significantly high in the deceased group as in previous studies [3–8,19,20]. 

In the univariate analysis, symptoms, expectoration, and anorexia; and comorbidities, cerebrovascular disease, dementia, chronic kidney disease, and malignancy were associated with mortality. Additionally, high values of APACHE II and SOFA scores, and CCI were related to 28-day mortality. In a previous study, dyspnea was defined as a risk factor for mortality [3], but, in this study, it did not differ between groups. A study from Wuhan revealed that anorexia is more frequent in nonsurvivor patients [22]. In previous studies, hypertension [7,8,22], diabetes mellitus [7], chronic heart disease [5,7], chronic lung disease [22], cerebrovascular disease [3,7], chronic kidney disease, and chronic liver disease [3], malignancy [5], and immunosuppression [7] were defined as risk factors for mortality. Although dementia and malignancy were significantly high in the deceased group, both comorbidities were not considered risk factors for 28-day survival in the multivariate analysis. There is still wide variability in the definition of comorbidities, symptoms, and signs as mortality risk factors in patients with COVID-19.

The data of the microbiological cultures showed that one or more positive blood or respiratory sample culture had a crucial impact on survival. Especially, a positive *Acinetobacter* spp. or *Klebsiella* spp. in tracheal aspirate sample culture was related to 28-day mortality. Lansbury et al. [23] evaluated 3834 COVID-19 pneumonia and showed *Acinetobacter* *baumannii* and *Klebsiella* *pneumoniae* in two and four patients, respectively. Compared with the low incidence of the prior data, a high incidence of these pathogens was notable in this study. Additionally, VAP was a major causative problem for death, but it was not an independent risk factor mortality in multivariate analysis.

According to the time of admission and recommendations in the national guideline, most patients received different antiviral agents. In univariate analysis, only favipiravir was used more commonly in the survivors in our cohort. There is still no evidence whether an antiviral agent has a beneficial effect on the recovery of COVID-19 [24]. The RECOVERY trial [25] revealed that dexamethasone and tocilizumab reduce mortality and the need for MV, but, in patients who receive corticosteroids, secondary infections may increase with delayed viral clearance [26–28]. Additionally, tocilizumab was considered shortening the discharge time [25]. In contrast, the use of both tocilizumab, and pulse/usual dose of corticosteroids did not reduce the risk of mortality in our study. This issue may be related to the retrospective design and small sample size. Also, we did not evaluate the discharge time and need for MV in patients receiving tocilizumab. Simonovich et al [29] showed that the use of convalescent plasma therapy in addition to standard of care did not reduce 30-day mortality in patients with severe pneumonia. In this study, the use of convalescent plasma therapy did not significantly differ between the groups.

Many kinds of respiratory support modalities revealed a significant effect on mortality. The need for NIV or IMV on hospital or ICU admission is associated with mortality compared with the COT or HFNC [8]. A study showed that survival was higher in patients who received COT, HFNC, or NIV compared with patients needed for IMV in the first 24 h [6]. Our data revealed that COT and HFNC were significantly high in the recovered patients, and patients who needed IMV had a high 28-day mortality rate. Additionally, in the laboratory data, the parameters related to severe respiratory failure including low oxygen saturation on ICU admission, hypoxemia, decreased global oxygen delivery, increased anaerobic metabolism, and acidosis were strongly associated with mortality. Likewise, a low PaO_2_ / FiO_2_ ratio on ICU admission was independently associated with mortality in a previous study [6]. Rapid progression of the disease, need for more advanced respiratory support may cause high mortality in patients with severe disease. However, delayed admission to ICU may be another reason for mortality, but, in this cohort, the duration from the first symptom to hospital or ICU admission did not differ between groups.

## 5. Limitations and strengths

This study has several limitations. First, the results are from a single center and should be supported by further studies. Second, acquired data was based on the retrospective evaluation of the records. We did not achieve some patients’ previous chest CT scans due to transfer from another center. Third, we could not analyze the long-term survival of the patients. However, the study has some strengths. We think that momentary data in ICUs for the conditions related to mortality provides a valuable data set. Second, reevaluation of the chest CT scans with a high interrater agreement, made this parameter more reliable.

## 6. Conclusion

In the present study, we revealed that sepsis/septic shock, AKI, acute cardiac injury, a CT score > 15 were the independent risk factors related to mortality in critically ill patients with COVID-19. Risk stratification in these patients, especially in the peak periods of the outbreak, is essential to the appropriate use of limited healthcare sources. Early prediction of the disease progression may contribute to this principle. It is also vital for patients who have a high risk of death. The results of the present study may improve the outcomes of the patients with COVID-19.

## Informed consent

The study protocol was approved by the Dokuz Eylül University Non-Interventional Research Ethics Committee (date: 09.11.2020 and number:2020/09-39), and informed consent was waived due to the retrospective nature of the study.
